# DMBT1 inhibition of *Pseudomonas aeruginosa* twitching motility involves its N-glycosylation and cannot be conferred by the Scavenger Receptor Cysteine-Rich bacteria-binding peptide domain

**DOI:** 10.1038/s41598-019-49543-w

**Published:** 2019-09-11

**Authors:** Jianfang Li, Stephanie J. Wan, Matteo M. E. Metruccio, Sophia Ma, Kamran Nazmi, Floris J. Bikker, David J. Evans, Suzanne M. J. Fleiszig

**Affiliations:** 1 School of Optometry, University of California, Berkeley, CA USA; 2 Vision Science Graduate Group, University of California, Berkeley, CA USA; 3 Department of Oral Biochemistry, Academic Centre for Dentistry Amsterdam, Free University of Amsterdam and University of Amsterdam, Amsterdam, The Netherlands; 4 College of Pharmacy, Touro University California, Vallejo, CA USA; 5 Graduate Groups in Microbiology and Infectious Disease & Immunity, University of California, Berkeley, CA USA

**Keywords:** Cellular microbiology, Infectious diseases

## Abstract

The scavenging capacity of glycoprotein DMBT1 helps defend mucosal epithelia against microbes. DMBT1 binding to multiple bacterial species involves its conserved Scavenger Receptor Cysteine-Rich (SRCR) domains, localized to a 16-mer consensus sequence peptide, SRCRP2. Previously, we showed that DMBT1 bound *Pseudomonas aeruginosa* pili, and inhibited twitching motility, a pilus-mediated movement important for virulence. Here, we determined molecular characteristics required for twitching motility inhibition. Heat-denatured DMBT1 lost capacity to inhibit twitching motility and showed reduced pili binding (~40%). Size-exclusion chromatography of Lys-C-digested native DMBT1 showed that only high-Mw fractions retained activity, suggesting involvement of the N-terminal containing repeated SRCR domains with glycosylated SRCR-Interspersed Domains (SIDs). However, individual or pooled consensus sequence peptides (SRCRPs 1 to 7) showed no activity and did not bind *P. aeruginosa* pili; nor did recombinant DMBT1 (aa 1–220) or another SRCR-rich glycoprotein, CD163. Enzymatic de-N-glycosylation of DMBT1, but not de-O-glycosylation, reduced its capacity to inhibit twitching motility (~57%), without reducing pili binding. Therefore, DMBT1 inhibition of *P. aeruginosa* twitching motility involves its N-glycosylation, its pili-binding capacity is insufficient, and it cannot be conferred by the SRCR bacteria-binding peptide domain, either alone or mixed with other unlinked SRCRPs, suggesting an additional mechanism for DMBT1-mediated mucosal defense.

## Introduction

DMBT1 (Deleted in Malignant Brain Tumors 1) is a ~340 kDa glycoprotein that was first isolated from saliva, belongs to the highly conserved Scavenger Receptor Cysteine-Rich (SRCR) protein superfamily, and is involved in mucosal innate immunity^1^. From the N-terminal, DMBT1 contains 13 highly homologous SRCR domains separated by SIDs (SRCR-interspersed domains). A 14^th^ SRCR domain is separated from the other 13 by a CUB (C1r/C1s Uegf Bmp1) domain, another of which separates the 14^th^ domain from the ZP (zona pellucida) domain that forms the C-terminal^2,3^. DMBT1 salivary agglutinin (DMBT1^SAG^) has been shown to interact with, and agglutinate, several Gram-positive and Gram-negative bacteria, except for *Pseudomonas aeruginosa*^4–8^. Similarly, tear fluid DMBT1 was shown to bind *Staphylococcus aureus*, but not *P. aeruginosa*^9^. DMBT1 binding of *Streptococcus mutans* and many other bacteria, *e.g. S. aureus*, *Escherichia coli*, and *Helicobacter pylori* involves the SRCR domains, and was specifically localized to a 16 amino acid consensus sequence peptide of DMBT1 designated SRCRP2^3^.

DMBT1 is expressed in multiple tissues and body fluids and can undergo modifications that affect its function at specific sites^1,9^. Indeed, there are different human DMBT1 alleles within the population and different isoforms in various tissues due to alternative splicing and post-translational modifications^1,4,10–12^. In salivary-derived DMBT1^SAG^, ~25% of the molecular mass is due to glycosylation (~10% for N-glycosylation, and ~15% for O-glycosylation)^13,14^.

Previously, we showed that tear fluid DMBT1, and DMBT1 purified from saliva, play a protective role for mucosal tissues by inhibiting twitching motility of *P. aeruginosa*^15^. Bacterial twitching motility is a surface-associated movement commonly used by Gram-negative bacteria driven by extension and retraction of Type IV pili (T4P)^16^. In *P. aeruginosa*, the T4P is a polymer predominately made up by the PilA subunit^17^. Extension and retraction, required for movement, is powered by ATPases PilB, PilU, and PilT^18^. In previous studies, we showed that *P. aeruginosa* twitching mutants were defective in host cell exit after cell invasion, exhibited impaired traversal of corneal epithelia *in vitro*^19^, and showed reduced virulence in the injured murine cornea^20^. Consistent with those results, in the study above^15^, purified DMBT1 inhibited *P. aeruginosa* from traversing multi-layered cultured epithelial cells *in vitro*, and reduced *P. aeruginosa* virulence in a murine model of corneal infection. It was also shown that DMBT1 could bind extracted pili from *P. aeruginosa* suggesting the involvement of pilus interaction in twitching inhibition.

Given the importance of twitching motility for *P. aeruginosa* virulence^20^, we sought to further understand mechanisms by which DMBT1 inhibits twitching motility, the relationship to pilus binding, and if the SRCR domains were involved. We report structure-function studies using purified salivary DMBT1 (DMBT1^SAG^), enzymatically-digested fragments, and synthetic DMBT1 consensus sequence peptides (SRCRP1–7) aimed at identifying the molecular domains and characteristics involved in mediating this host defense against *P. aeruginosa*.

## Results

### DMBT1 inhibition of *P. aeruginosa* twitching motility is lost and pili binding reduced after heat denaturation

**Figure 1:**
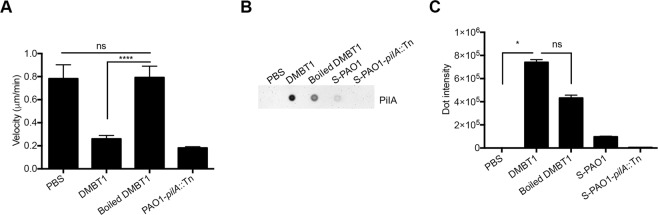
Boiled DMBT1 does not inhibit *P. aeruginosa* twitching motility. DMBT1 (100 ng/µL) was heated at 95 °C for 10 min to denature the protein. **(A)** Boiled DMBT1 lost inhibitory activity against *P. aeruginosa* PAO1 twitching velocity. Data are shown as the mean ± SEM per sample from three independent experiments. One-way ANOVA with Tukey’s post-hoc analysis, ****P < 0.0001; ns, not significant. **(B)** Dot-immunoblot assay using anti-PilA antibody to show binding of PAO1 pili to DMBT1 (500 ng) or boiled DMBT1 (500 ng) after 40 min incubation with a pili-containing extract from PAO1. Diluted pili extracts from PAO1 (S-PAO1) or similarly diluted extracts from its *pilA* mutant (S-PAO1-*pilA*::Tn) served as controls (see Methods), along with PBS alone. A representative experiment of two independent experiments is shown. **(C)** Quantification of dot-intensity from the dot-immunoblot assay shown in panel B. Data are shown as the mean ± SEM of triplicate measurements from each sample. Kruskal-Wallis test with Dunn’s multiple comparisons, *P < 0.05; ns, not significant.

Previously, we showed that DMBT1 purified from human saliva inhibited *P. aeruginosa* (PAO1) twitching motility and could also bind to extracted pili^15^. To determine if these activities were heat-stable, purified salivary glycoprotein DMBT1 was boiled for 10 min at 95 °C. This protein-denaturing treatment abrogated DMBT1 inhibition of twitching motility (as measured by twitching velocity) (Fig. 1A) suggesting that glycoprotein conformation is important for this activity. Furthermore, the anti-PilA dot-blot and its quantification showed that boiled DMBT1 exhibited reduced ability to bind extracted pili (~40%) compared to untreated DMBT1 (Fig. 1B,C). Although the reduction of pili binding after heat denaturation was not statistically significant, the data suggest that DMBT1 binding to pilin is optimal in its native conformation.

### Lys-C endoproteinase digestion of DMBT1 does not abolish inhibition of twitching motility

**Figure 2:**
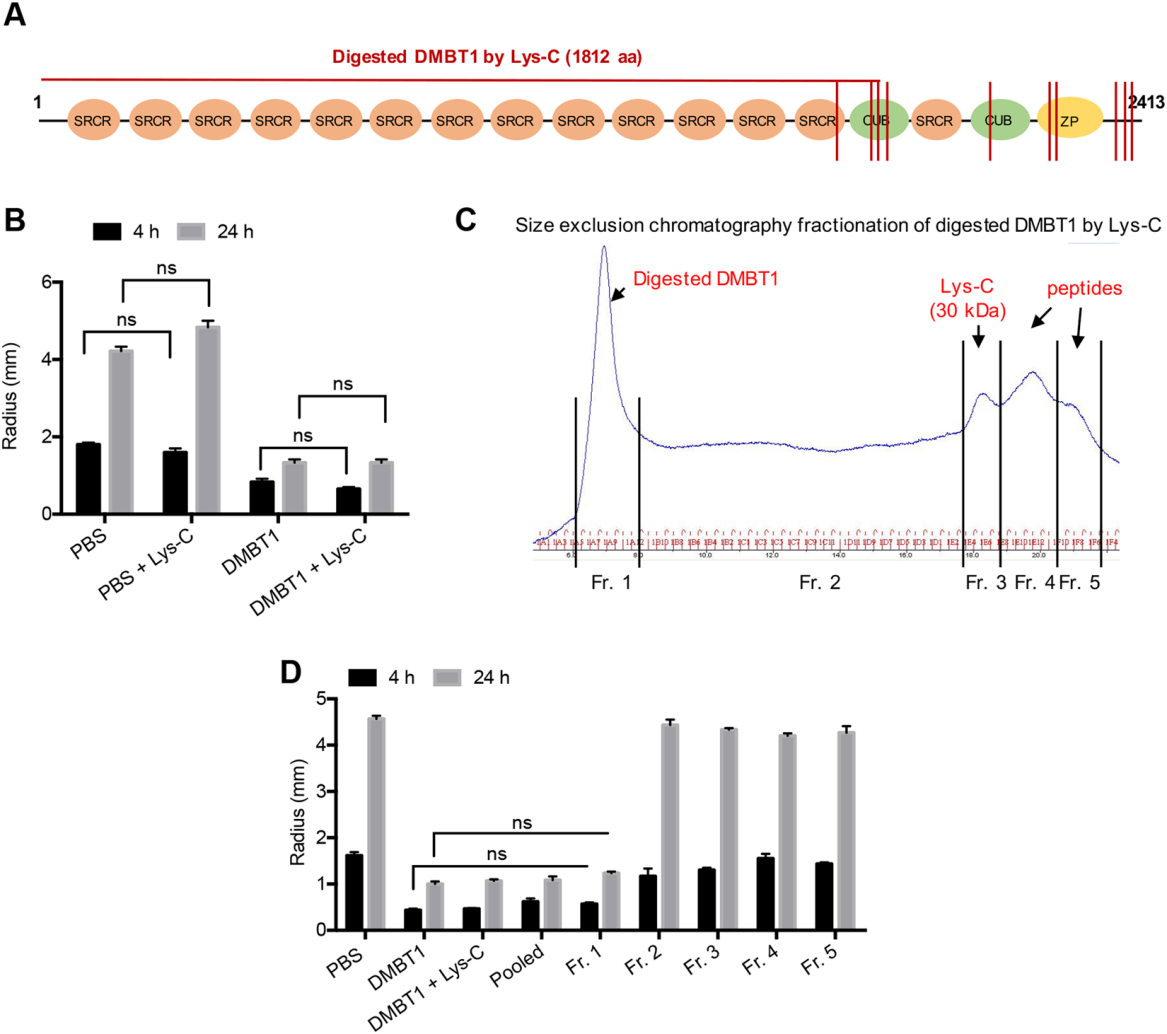
DMBT1 digested by Lys-C under native conditions inhibits *P. aeruginosa* twitching motility. (**A**) Schematic representation of human salivary DMBT1 with 14 scavenger receptor cysteine-rich (SRCR) domains, 2 CUB domains, and the Zona Pellucida (ZP) domain, showing endoproteinase Lys-C cleavage sites (red lines). (**B**) Effect of Lys-C digested DMBT1 (100 ng/µL) versus a DMBT1 control on colony size of *P. aeruginosa* strain PAO1 after 4 h and 24 h incubation. Two-way ANOVA with Tukey’s multiple comparisons, ns, not significant. **(C)** Size exclusion chromatography separation of Lys-C digested DMBT1. Fraction 1 contains high molecular-weight fragments (including aa 1–1812). **(D)** Colony size of *P. aeruginosa* after 4 h and 24 h incubation in different fractions of Lys-C digested DMBT1. Only fraction 1 inhibited *P. aeruginosa* twitching motility, similar to DMBT1. Two-way ANOVA with Tukey’s multiple comparisons, ns, not significant.

To further investigate the role of different DMBT1 domains in the inhibition of *P. aeruginosa* twitching motility, purified salivary DMBT1 was enzymatically digested with Lys-C, an endoproteinase. Others have shown that DMBT1 contains 10 lysine residues located in the C-terminal region^3^. As such, Lys-C digestion should produce a large fragment containing the majority of SRCR domains and SRCR Interspersed Domains (SIDs) in addition to several smaller fragments (Fig. 2A). Lys-C endoproteinase digested DMBT1 retained inhibition of *P. aeruginosa* twitching motility (Fig. 2B). Size exclusion chromatography of Lys-C digested DMBT1 under native buffer conditions showed an expected pattern of fractionation (Fig. 2C). Each Lys-C digested DMBT1 fraction obtained under native buffer conditions was tested for inhibition of *P. aeruginosa* twitching motility. Only Fraction 1, expected to contain fragments of combined high molecular-weight SRCR/SID domains (aa 1–1812), along with intact DMBT1, inhibited bacterial twitching motility (Fig. 2D), suggesting that the SRCR/SID components of DMBT1 were required, and showing that smaller fragments of the C-terminal region produced by Lys-C digestion were ineffective.

### Evaluation of DMBT1 SRCR domains, its N-terminal peptide, and CD163

**Figure 3:**
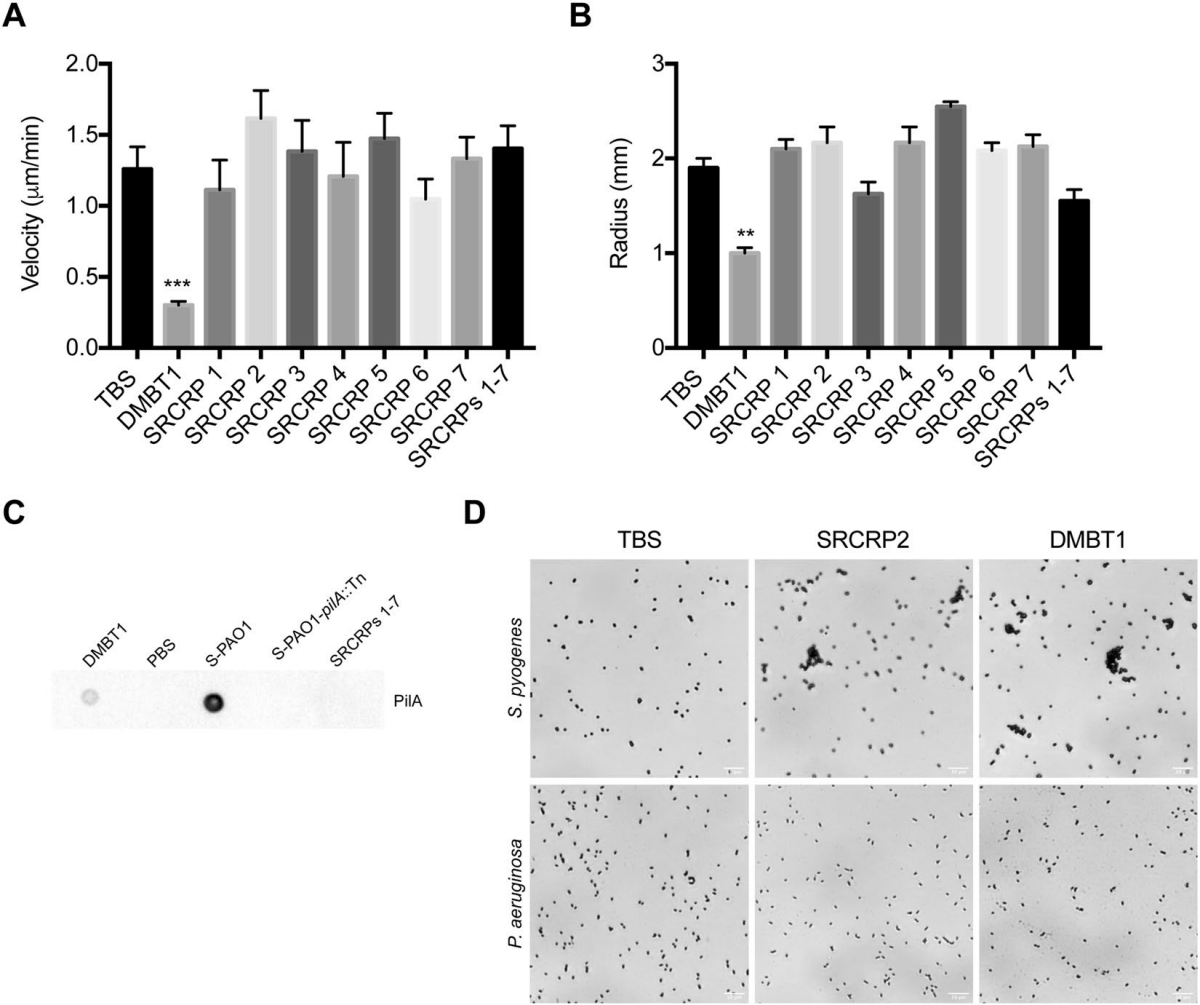
SRCR domain consensus sequence peptides do not inhibit twitching motility. Effect of custom-synthesized peptides SRCRPs 1–7 (200 ng/µL diluted in TBS with 10 mM CaCl_2_) on **(A)** twitching velocity, and **(B)** colony size, of *P. aeruginosa* strain PAO1 after 4 h. Data are shown as the mean ± SEM. Experiments were performed in triplicate and significance determined using one-way ANOVA with Dunnett’s multiple comparisons test. ***P < 0.001, **P < 0.01. None of the peptides showed a significant difference versus the TBS (with 10 mM CaCl_2_) control. **(C)** Dot-immunoblot assay using anti-PilA antibody shows the binding of PAO1 pili to purified DMBT1 (400 ng), but not to pooled SRCRPs 1–7 (400 ng). Diluted pili extracts from PAO1 (S-PAO1) or those from its *pilA* mutant (S-PAO1-*pilA*::Tn) and PBS were used as controls. **(D)** Agglutination assay showing that purified salivary DMBT1, and the consensus sequence peptide SRCRP2, agglutinated/aggregated *S. pyogenes* (upper panels) as previously shown^19^, but not *P. aeruginosa* (lower panels), versus TBS controls (see Methods).

Fraction 1 of Lys-C digested DMBT1 should have contained high-molecular weight fragments consisting of combined multiple SRCR/SID domains (aa 1–1812). Thus, a series of custom-synthesized peptides (SRCRP) spanning the consensus sequence of the DMBT1 SRCR domains^3^ were tested. Indeed, SRCRP2 has previously been shown to be involved in bacterial binding^3^. However, each of the individual SRCR consensus-based peptides tested (SRCRPs 1–7) had no effect on *P. aeruginosa* twitching motility (Fig. 3A,B), and no inhibition was observed when these peptides were pooled (Fig. 3A,B). It was also confirmed that (pooled) SRCR peptides (1–7) did not bind pili (Fig. 3C). A control experiment confirmed previous findings that SRCRP2 caused agglutination/aggregation of *Streptococcus* spp. (shown here using *S. pyogenes*)^3^, but not *P. aeruginosa* (Fig. 3D). As we have shown previously^15^, purified salivary DMBT1 inhibited *P. aeruginosa* twitching motility (Fig. 3A,B), but did not cause bacterial agglutination/aggregation (Fig. 3D). Thus, exposing *P. aeruginosa* to the consensus sequence of the DMBT1 SRCR domains (albeit in fragments) was insufficient to inhibit twitching motility, or to bind extracted pili, supporting our previous hypothesis^15^ that DMBT1 inhibition of *P. aeruginosa* twitching motility involves a different type of bacterial cell interaction than shown for other bacterial species^3^.

**Figure 4:**
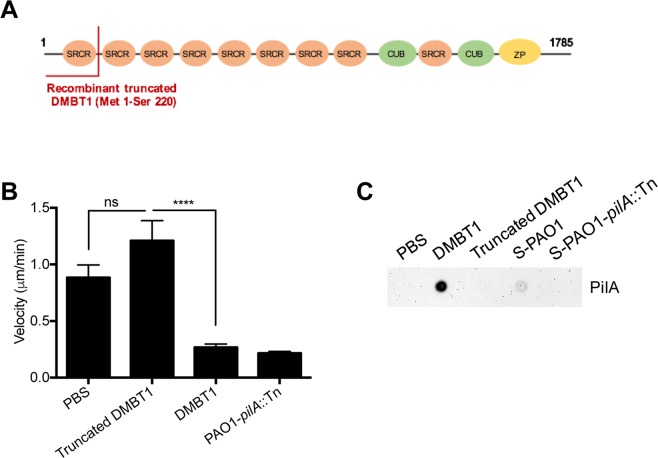
Recombinant DMBT1 (Met 1 - Ser 220) has no effect on *P. aeruginosa* twitching motility. **(A)** Schematic representation of recombinant truncated human DMBT1 (NP_004397.2) (Met 1-Ser 220) shown in the primary sequence of DMBT1 with 9 scavenger receptor cysteine-rich (SRCR) domains, 2 CUB domains and Zona pellucida (ZP) domain. **(B)** Effect of truncated DMBT1 (100 ng/µL) on twitching velocity of *P. aeruginosa* strain PAO1 after 4 h. PAO1-*pilA*::Tn served as a negative control. One-way ANOVA with Tukey’s multiple comparisons test, ****P < 0.0001. **(C)** Dot-immunoblot assay using anti-PilA antibody shows binding of extracted PAO1 pili to DMBT1 (500 ng), but not truncated DMBT1 (500 ng). Pili extracts from PAO1 (S-PAO1), extracts from the *pilA* mutant (S-PAO1-*pilA*::Tn), both diluted 1 in 500 in PBS were used as controls, along with PBS alone.

Since Fraction 1 of Lys-C digested DMBT1 would also contain the N-terminal region (albeit as part of a large fragment containing multiple SRCRs/SIDs), a recombinant truncated peptide consisting of the N-terminal domain of DMBT1 (Met1-Ser 220) (Fig. 4A) was tested. However, this recombinant N-terminal peptide had no effect on twitching motility (Fig. 4B), and did not bind pili (Fig. 4C).

**Figure 5:**
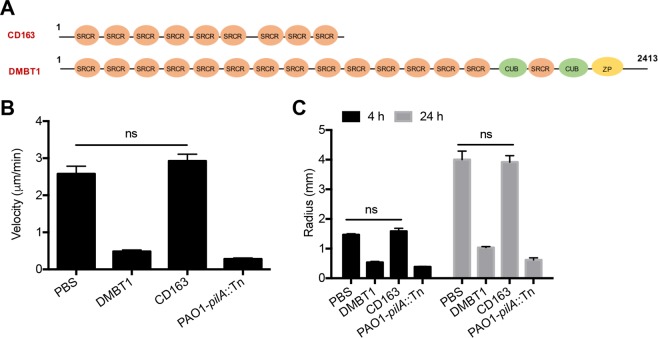
Glycoprotein CD163 does not inhibit *P. aeruginosa* PAO1 twitching motility. (**A)** Schematic representation of CD163 as expressed in HEK 293 cells (BioVision, Inc.) compared to human salivary DMBT1. **(B)** Effect of CD163 (200 ng/µL) on twitching velocity of *P. aeruginosa* PAO1 after 4 h treatment (One-way ANOVA with Tukey’s multiple comparisons, ns, not significant), and **(C)** on colony size after 4 h and 24 h. PAO1-*pilA*::Tn served as a negative control. Significance determined using two-way ANOVA with Tukey’s multiple comparisons, ns, not significant.

CD163 was also tested for inhibition since it is a member of the SRCR-superfamily (Fig. 5A). Moreover, this plasma membrane glycoprotein is expressed on monocytes and macrophages and functions as a receptor for bacteria, resulting in the expression of proinflammatory cytokines^21^. However, results showed that recombinant human CD163 had no effect on *P. aeruginosa* twitching motility (Fig. 5B,C), despite considerable similarities in structure, *i.e*. multiple SRCR/SID regions, to DMBT1.

### N-glycosylation of DMBT1 is important for its inhibition of *P. aeruginosa* twitching motility

**Figure 6:**
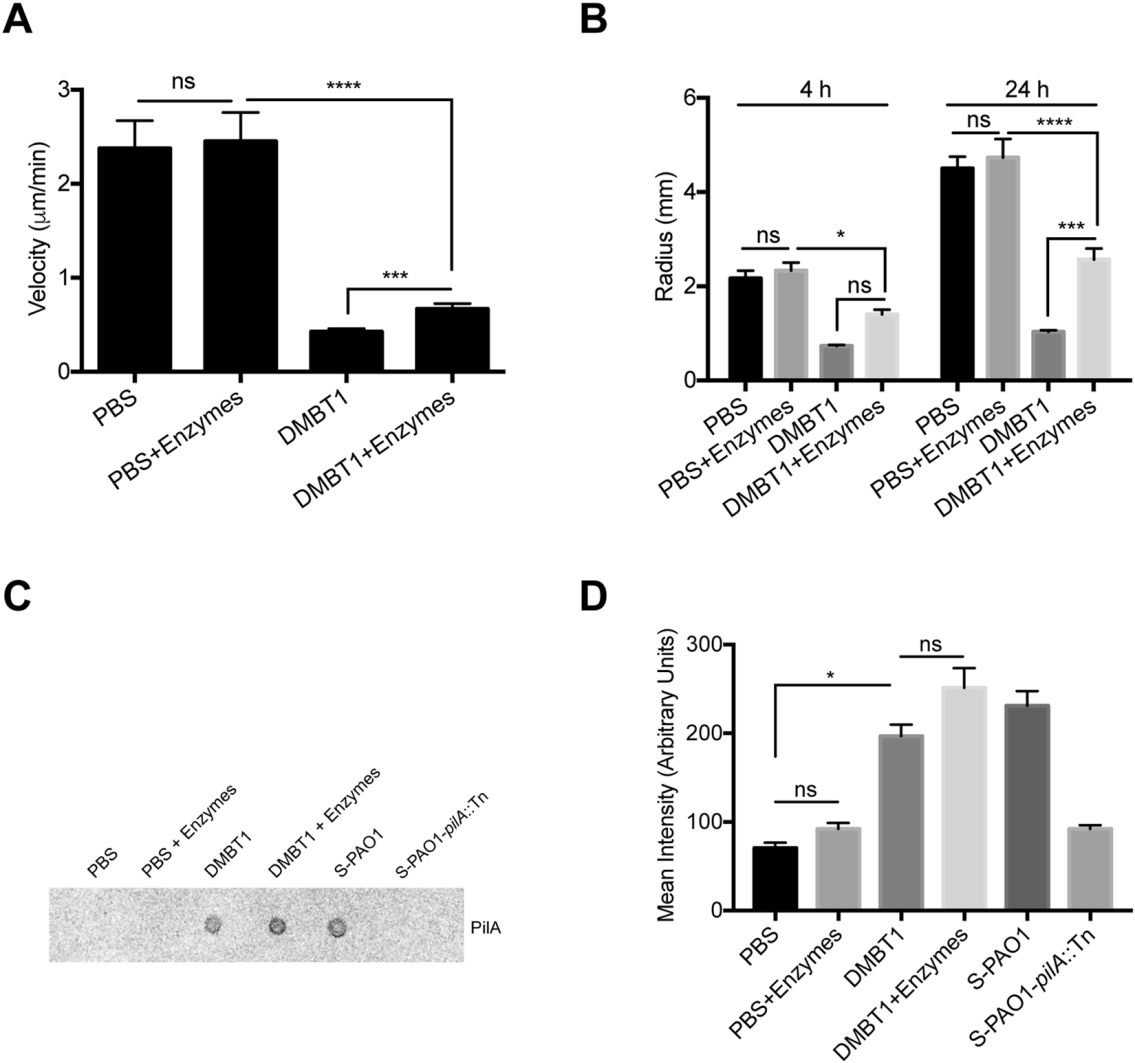
DMBT1 glycosylation is involved in inhibition of *P. aeruginosa* twitching motility. Effect of DMBT1 digested by deglycosylation enzymes on **(A)** twitching velocity of *P. aeruginosa* PAO1 after 4 h, and **(B)** colony size of *P. aeruginosa* PAO1 after 4 h and 24 h. Data are shown as the mean ± SEM per sample from three independent experiments. Significance was determined using one-way ANOVA (panel A) or two-way ANOVA (panel B) each with Tukey’s multiple comparisons, ****P < 0.0001; ***P < 0.001; *P < 0.05; ns, not significant. (**C)** Dot-immunoblot assay with anti-PilA antibody shows continued binding of PAO1 pili to purified DMBT1 (400 ng) after deglycosylation digestion. Pili extracts from PAO1 (S-PAO1), extracts from the *pilA* mutant (S-PAO1-*pilA*::Tn), both diluted 1 in 500 in PBS were used as controls, along with PBS alone. **(D)** Quantification of dot-intensity using ImageJ from the dot-immunoblot assay shown in panel C. Data are shown as the mean ± SEM of duplicate measurements from each sample. Significance determined used Kruskal-Wallis test with Dunn’s multiple comparisons, *P < 0.01; ns, not significant.

Having established that inhibition of twitching motility resided in high molecular-weight fractions of Lys-C digested DMBT1 containing the N-terminal region and combined SRCR/SID domains (aa 1–1812), but without finding activity on individual SRCR peptides of DMBT1 or proteins with similar SRCR composition, we studied if post-translational modifications were involved. Since DMBT1 is a highly-glycosylated protein^2^, we postulated that glycosylation was involved in twitching inhibition. To test this, DMBT1 was digested by a deglycosylation enzyme mix containing N-glycosidase PNGase F, O-glycosidase, neuraminidase, exoglycosidase β1-4 galactosidase, and β-N-acetylglucosaminidase under native conditions to remove all N-linked and simple O-linked glycans, as well as some complex O-linked glycans (see Methods). After deglycosylation, DMBT1 showed significantly reduced inhibition activity of *P. aeruginosa* twitching motility compared to native DMBT1 controls, as measured by twitching velocity (*P* < 0.001, Fig. 6A) and colony size (*P* < 0.001 at 24 h, Fig. 6B). Examination of colony size data at 24 h indicated that full O- and N deglycosylation of DMBT1 reduced its ability to inhibit twitching motility by ~41% (Fig. 6B). However, deglycosylated DMBT1 still bound to pili as shown by dot-immunoblot assay (Fig. 6C,D). These results suggest that the glycosylation of DMBT1 is involved in the inhibition of *P. aeruginosa* twitching motility. However, observation that reduced DMBT1-mediated twitching inhibition did not correlate with any reduction in pili binding suggests that additional (or other) mechanisms may be involved.

**Figure 7:**
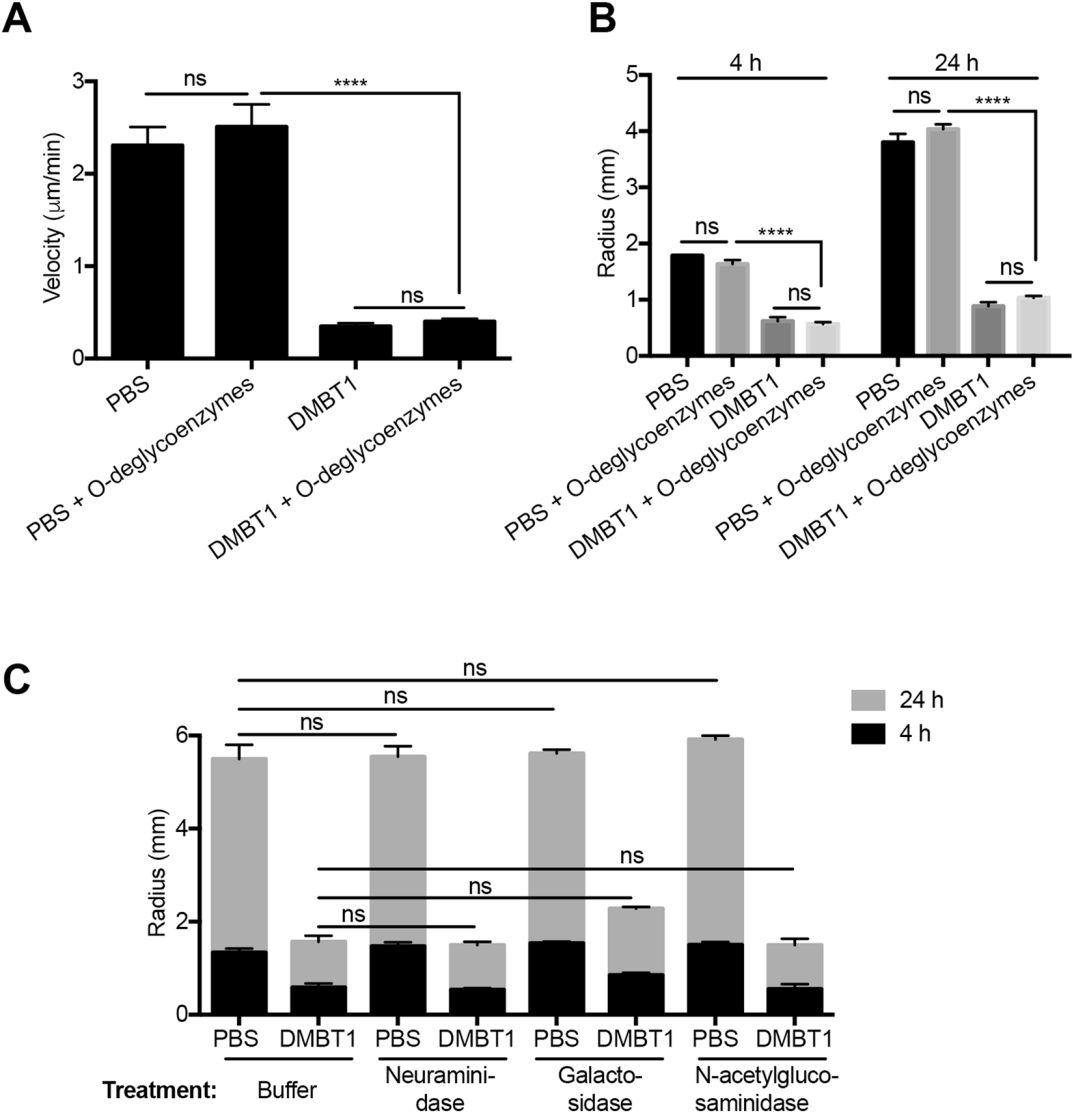
O-Glycosylation of DMBT1 does not contribute to inhibition of bacterial twitching motility. (**A**) Effect of DMBT1 digested by an O-deglycosylation enzyme mix on twitching velocity of *P. aeruginosa* PAO1 after 4 h. DMBT1 (100 ng/µL) was digested by a mixture of O-deglycosylation enzymes (O-glycosidase, Neuraminidase, Galactosidase and N-acetyl-glucosaminidase) at 37 °C for 2 days in native glycobuffer (50 mM sodium phosphate, pH 7.5) to remove complex O-glycosylation of DMBT1. PBS and DMBT1 diluted with same volume of glycobuffer served as controls. **(B)** Effects of DMBT1 digested by the O-deglycosylation enzyme mix on colony size of *P. aeruginosa* PAO1 after 4 h and 24 h. **(C)** Effects of differently digested DMBT1 on colony size of *P. aeruginosa* PAO1 after 4 h and 24 h. DMBT1 (100 ng/µL) was digested by Neuraminidase (desialylation glycosidase), Galactosidase or N-acetylglucosaminidase (both exoglycosidases) at 37 °C for 2 h in glycobuffer (50 mM sodium acetate, 0.5 mM CaCl_2_, pH 5.5) to remove sialic acid residues and other complex glycosylation of DMBT1. PBS and DMBT1 diluted with same volume of glycobuffer served as controls. Data shown as the mean ± SEM per sample from three independent experiments. Significance was determined using one-way ANOVA (panel A) or two-way ANOVA (panel B) each with Tukey’s multiple comparisons, ****P < 0.0001; ns, not significant.

We next explored if the type of DMBT1 glycosylation linkage was important by selectively targeting O-linked versus N-linked moieties. The involvement of O-glycosylation in DMBT1 inhibition of twitching activity was studied with an O-glycan targeted enzyme mix (O-glycosidase, neuraminidase, exoglycosidase β1-4 galactosidase, and β-N-acetylglucosaminidase) under native conditions. No significant difference was found between DMBT1 and de-O-glycosylated DMBT1 on twitching inhibition (Fig. 7A,B). The same results were obtained using longer incubation times with bacteria (from 24 to 48 h), and/or higher concentrations of deglycosylation enzymes (data not shown). According to the manufacturer’s (Biolabs) documentation, this combination of enzymes may not remove all O-linked oligosaccharides, but should remove many common oligosaccharide structures under native conditions. Since denatured DMBT1 lost inhibition of twitching motility (Fig. 1A), denatured de-O-glycosylation could not be used to completely remove O-linked glycans. Nevertheless, the results suggest that simple O-glycosylation is not involved in DMBT1 inhibition of *P. aeruginosa* twitching motility.

To study if complex DMBT1 O-glycosylation could be involved in twitching motility inhibition, DMBT1 was digested with neuraminidase, β1-4 galactosidase, or β-N-acetylglucosaminidase in a different (more optimal) digestion buffer (50 mM sodium acetate, 0.5 mM CaCl_2_, pH 5.5). However, none of these complex de-O-glycosylation treatments of DMBT1 had a significant effect on its ability to inhibit twitching motility, although β1-4 galactosidase appeared to have some effect (Fig. 7C). Thus, the data suggest that complex O-glycosylation of DMBT1 was also not required for inhibition of twitching motility.

**Figure 8:**
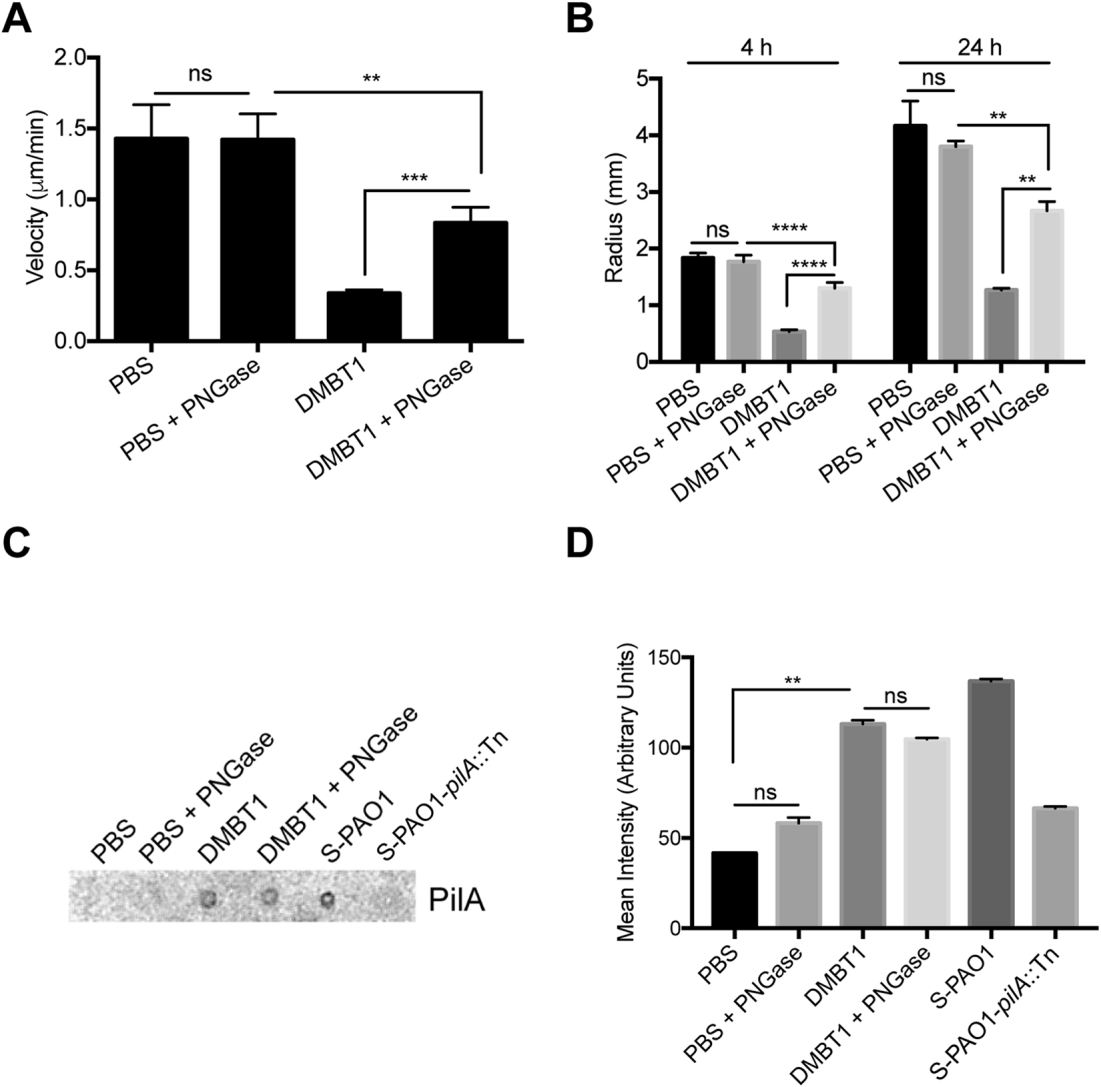
DMBT1 inhibition of *P. aeruginosa* twitching motility involves its N-Glycosylation. DMBT1 (100 ng/µL) was digested by PNGase F (glycerol-free) at 37 °C for 2 days in native glycobuffer (50 mM sodium phosphate, pH 7.5) to remove N-glycosylation of DMBT1. PBS and DMBT1 diluted with same volume of native glycobuffer, and PBS treated with same volume of PNGase served as controls. **(A)** Effect of DMBT1 digested by PNGase F on twitching velocity of *P. aeruginosa* PAO1 after 4 h. **(B)** Effects of DMBT1 digested by PNGase F on colony size of *P. aeruginosa* PAO1 after 4 h and 24 h. Data are shown as the mean ± SEM per sample from three independent experiments. Significance was determined using one-way ANOVA (panel A) or two-way ANOVA (panel B) each with Tukey’s multiple comparisons, ****P < 0.0001; ***P < 0.001; **P < 0.01; ns, not significant. **(C)** Dot-immunoblot assay using anti-PilA antibody showing the binding of PAO1 pili to purified DMBT1 (400 ng) even after PNGase F digestion. Pili extracts from PAO1 (S-PAO1), extracts from the *pilA* mutant (S-PAO1-*pilA*::Tn), both diluted 1 in 500 in PBS were used as controls, along with PBS alone. **(D)** Quantification of dot-intensity using ImageJ from the dot-immunoblot assay shown in panel C. Data are shown as the mean ± SEM of duplicate measurements from each sample. Significance determined used Kruskal-Wallis test with Dunn’s multiple comparisons, **P < 0.01; ns, not significant.

Purified DMBT1 was digested with PNGase F as well in order to remove N-linked glycans in native buffer. De-N-glycosylation enzyme treatment of DMBT1 significantly reduced its ability to inhibit *P. aeruginosa* twitching motility compared to DMBT1 (Fig. 8A,B). Examination of colony size data at 24 h indicated that de-N-glycosylation of DMBT1 reduced its ability to inhibit twitching motility by ~57% (Fig. 6B). Thus, the extent to which DMBT1 de-N-glycosylation reduced twitching motility inhibitory activity versus untreated DMBT1 was similar to, if not greater than, that observed with non-specific deglycosylation (Fig. 6A,B). Thus, these data suggest that N-glycosylation of DMBT1 is involved in the inhibition of twitching motility. However, de-N-glycosylated DMBT1 still showed a significant inhibition of twitching motility compared to PBS-enzyme controls (Fig. 8A,B), suggesting that N-glycosylation of DMBT1 is only partly involved in twitching inhibitory activity, and other molecular characteristics are also required. Furthermore, de-N-glycosylated DMBT1 still bound to pili similarly to native DMBT1 (Fig. 8C,D), suggesting that factors additional to, or instead of, direct pilus interaction are needed.

## Discussion

Previously, we showed that the glycoprotein DMBT1 in human tear fluid, and purified from human saliva, inhibited twitching motility of *P. aeruginosa* and consequently prevented bacterial traversal through human corneal epithelial cells *in vitro*, and reduced disease in a murine model of corneal infection^15^. Here, we focused on determining how salivary DMBT1 (DMBT1^SAG^) inhibited twitching motility using structure-function studies. Results showed that the ability of salivary DMBT1 to inhibit twitching motility was removed by heat denaturation, correlating with a reduction in binding to purified pili. Results also showed that Lys-C digestion of DMBT1 had no effect on twitching inhibition, the activity residing in high molecular-weight fractions after Lys-C digestion. Moreover, no effect on twitching inhibition was observed for custom-synthesized individual, or pooled, SRCR-consensus-sequence peptides, recombinant N-terminal DMBT1, nor the SRCR-SID rich glycoprotein CD163. However, experiments using enzymatic deglycosylation of DMBT1 showed that the inhibition of twitching motility was partially, but significantly, associated with N-glycosylation, but not O-glycosylation, and that loss of inhibitory activity after de-N-glycosylation did not correlate with a reduction in binding to purified pili.

The finding that heat denatured salivary DMBT lost its ability to inhibit *P. aeruginosa* twitching motility suggests that structural conformation is important for this activity. Indeed, the result is consistent with our previous results with tear fluid containing DMBT1, which also lost its inhibitory effects on twitching motility after heat denaturation^15^. In addition to loss of structural conformation, loss of inhibitory activity after heat denaturation could include the compromise of DMBT1 moieties that normally bind bacterial ligands. Indeed, heat denaturation did cause reduced DMBT1 binding to pili extracts from *P. aeruginosa*. Heat denaturation may also cause a loss of DMBT1-associated molecules that mediate, or help-mediate, twitching inhibition. However, in our previous study, mass spectrometry analysis of purified salivary DMBT1 did not reveal any known DMBT1-associated innate defense molecules^15^, suggesting this possibility is less likely.

Digestion of saliva-purified DMBT1 with Lys-C followed by size exclusion chromatography, suggested that the SRCR/SID domains of salivary DMBT1 were essential for the inhibition of *P. aeruginosa* twitching motility, *i.e*. high-Mw fragments of the N-terminal (aa 1–1812). That result would be consistent with a previous study in which Lys-C digestion of salivary DMBT1 revealed that binding of *S. mutans* was associated with SRCR/SID domains^3^. In that study, and a subsequent study, a series of custom-synthesized peptides that together span the 109 amino acid consensus sequence of the SRCR domains of salivary DMBT1 were used to identify specific bacterial binding regions. SRCR-mediated DMBT1 binding of *S. mutans* and many other bacteria, *e.g. Staphylococcus aureus*, *Escherichia coli*, and *Helicobacter pylori*, was specifically localized to a 16 amino acid peptide designated SRCRP2, and even more specifically to a minimal bacterial binding region of 11 amino acids (the DMBT1 pathogen-binding site)^3,22^. In our study, however, SRCRP2 did not inhibit *P. aeruginosa* twitching motility, nor did it bind pili extracts. Controls also confirmed that SRCRP2 could agglutinate *Streptococcus* spp. as previously shown^3^, but not *P. aeruginosa*. Moreover, none of the other SRCR peptides representing other regions of the SRCR domain consensus sequence had any effect either individually or pooled. Of course, these findings do not preclude involvement of the SRCR domains in DMBT1-mediated inhibition of *P. aeruginosa* twitching motility, *e.g*. use of peptides with a different sequence from the SRCR domain consensus sequence may be needed. Alternatively, SID domains may be required by themselves, or to bridge a series of SRCR domains. For example, a region of the N-terminal domain of DMBT1 containing one SRCR and half of its neighboring SID binds HIV-1 gp-120 to exert antiviral effects^23^. Moreover, similar molecules to DMBT1 with multiple, connected SRCR domains*, e.g*. CD163 expressed on tissue macrophages, also bind multiple different bacteria triggering release of proinflammatory mediators^21^. However, neither recombinant N-terminal DMBT1 (aa 1–220) nor recombinant CD163 had any effect on *P. aeruginosa* twitching, nor did they bind pili extracts. Again, this does not preclude an involvement of the N-terminal domain of DMBT1 with multiple SRCR domains and their respective SIDs, in the inhibition of *P. aeruginosa* twitching motility. However, these collective findings do suggest that the mechanism involves DMBT1-bacterial cell interaction(s) that differ from those reported for numerous other Gram-positive and Gram-negative bacteria, at least it cannot be conferred by the SRCR bacteria-binding peptide domain alone, or when mixed (albeit unlinked) with other consensus sequence peptides.

It is possible that the activity of high-Mw fractions of Lys-C digested DMBT1 reflect the presence of residual undigested DMBT1, since it is very difficult to separate undigested native DMBT1 from other high-Mw fragments, and heat denaturation removed its twitching inhibitory activity. Even if residual DMBT1 were present, however, that would not change the above conclusion that the mechanism(s) of twitching motility inhibition likely involve a different type of DMBT1 interaction with *P. aeruginosa* than reported for other bacteria. Moreover, lack of activity of smaller-Mw fractions of Lys-C digested DMBT1 does show that the C-terminal fragments generated by Lys-C digestion were, by themselves, insufficient for twitching inhibition. That finding, in turn, suggests that twitching inhibition does not involve the two CUB domains and ZP domain of DMBT1.

Enzymatic deglycosylation of salivary DMBT1 did result in a partial reduction in twitching motility, which appeared to be specifically associated with the removal of N-glycosylated residues (~46% reduction in DMBT1 effects on bacterial twitching velocity, and a ~57% reduction in effects on bacterial colony size). While only a partial reduction in twitching inhibitory effects, the data show that N-glycosylation of DMBT1 is important for inhibition of *P. aeruginosa* twitching motility. N-linked glycosylation is known to be critical for general protein stability and dynamics^24–26^. Indeed, the precise location of N-glycans are significant for activity^24^. This could help to explain why CD163, a SRCR/SID containing bacterial receptor protein very similar to DMBT1 with many N-glycans, did not inhibit twitching of *P. aeruginosa*. For respiratory mucosal fluid-derived DMBT1 (gp-340) there are 14 potential sites of N-linked glycosylation^2^, only 4 of which reside within the N-terminal fragment of DMBT1 formed after Lys-C digestion. However, despite our data implicating SRCR/SID domains of DMBT1 (aa 1–1812) in twitching motility inhibition, and the absence of effects of C-terminal DMBT1 fractions obtained after Lys-C digestion (Fig. 2), it remains possible that C-terminal N-glycosylation (9 sites between aa 1813–2413) could also play a role. Interestingly, while not specific to DMBT1, N-glycans of tear proteins have been implicated in mediating their binding to clinical ocular isolates of *P. aeruginosa*^27^. As such, further investigation is warranted to determine which DMBT1 N-glycosylation site(s) is/are important for the twitching inhibitory phenotype.

While glycosylation differences (*e.g*. in sialyl-Le [Lewis] antigens) have been noted between different isoforms of DMBT1 (gp-304) from tear fluid, saliva, and respiratory fluid^28,29^, we previously noted that sialyl-Le (Lewis) antigen differences between DMBT1 isoforms would unlikely be involved since both tear fluid and salivary DMBT1 inhibit twitching motility, but express different sialyl-Le antigens^15^. Moreover, sialyl-Le antigens are attached to tear fluid and salivary DMBT1 by O-glycosylation^29,30^, and in the present study, enzymatic de-O-glycosylation of DMBT1 did not impact its inhibition of *P. aeruginosa* twitching motility.

While de-N-glycosylation reduced DMBT1’s ability to inhibit twitching, there remained a significant inhibition versus PBS indicating that other components of DMBT1 also contribute to twitching inhibition. Since heat denaturation of DMBT1 resulted in a complete loss of activity (Fig. 1), these other mechanisms are also heat-sensitive. However, given the similarity of inhibition levels between de-N-glycosylation and general deglycosylation (Fig. 8B versus Fig. 6B), and the lack of effect of specific de-O-glycosylation (Fig. 7B), suggest that these other mechanisms of twitching inhibition relate to other aspects of DMBT1 molecular structure.

In our previous study, the ability of DMBT1 to inhibit twitching motility correlated with binding to extracted bacterial pili suggesting involvement in the inhibitory mechanism^15^. In the present study, however, this correlation was weakened by two observations; (1) that heat-denatured DMBT1 retained pili binding (~60% after denaturation) despite the complete loss of activity, and (2) that de-N-glycosylation had no effect on pili binding, yet inhibitory activity was reduced by ~57%. Thus, DMBT1 can bind *P. aeruginosa* pili without inhibiting twitching motility. Previous studies have shown that DMBT1 (gp-340) can bind bacterial pili with various outcomes. For example, pili-mediated binding of Group A Streptococci to gp-340 was associated with bacterial aggregation and defense against bacterial adhesion^31^. In contrast, pili-mediated binding of Group B Streptococci to surface immobilized gp-340 was proposed as a host colonization mechanism, while bacterial aggregation by fluid-phase gp-340, a likely host defense, was pili independent^5^. Thus, our findings do not preclude an involvement of pili binding in DMBT1-mediated inhibition of twitching motility as a host defense, but suggest that this activity cannot be accomplished by binding to pili alone. Alternatively, DMBT1 binding to *P. aeruginosa* pili may serve an entirely separate function from twitching motility inhibition, either contributing to host defense *via* an additional mechanism or even to bacterial pathogenesis if circumstances allow, *e.g*. under a contact lens. Other potential mechanisms for DMBT1 inhibition of *P. aeruginosa* twitching motility could involve the deregulation of gene expression in the Pil-Chp pathway that controls type IV pilus production and twitching motility^32,33^. While our previous study showed that genes *cyaB, chpB*, and *pilK* were not necessary for DMBT1-mediated twitching inhibition^15^, other genes in this pathway could still be affected. DMBT1 could also interact with other proteins involved in pilus extension or retraction (*e.g*. ATPases PilB, PilT and PilU or chemosensory protein PilJ)^34–36^ which could compromise twitching motility. Thus, the role of DMBT1-pili interactions in the inhibition of *P. aeruginosa* twitching motility may be complex, and understanding them may require significant further study.

In conclusion, the results of this study add to our understanding of DMBT1-mediated inhibition of *P. aeruginosa* twitching motility, a contribution to mucosal fluid defense of surface epithelia that would help prevent bacteria from traversing mucosal epithelia, forming surface-associated biofilms, and causing disease pathology. The data show that the inhibitory mechanism of human salivary DMBT1 (DMBT1^SAG^) is heat-sensitive, suggesting that molecular conformation is important, and that DMBT1 N-glycosylation, but not O-glycosylation, contributes to ~50% or more of twitching inhibitory activity. The results also show that salivary DMBT1 can bind *P. aeruginosa* pili without inhibiting twitching motility, suggesting that if pili binding is involved, additional mechanisms are likely to play a role. Nevertheless, the lack of twitching inhibitory activity of consensus sequence-based peptides of salivary DMBT1, and presence of twitching inhibitory activity despite enzymatic de-O-glycosylation, suggest that the mechanism(s) involved differ from those demonstrated for DMBT1-mediated defense against other bacterial pathogens.

## Materials and Methods

### Bacterial strains and culture conditions

*P. aeruginosa* strain PAO1 was used for this study. Bacteria were grown on tryptic soy agar (TSA) plates (37 °C, 16 h) to obtain “lawn” cultures. The pilin mutant PAO1*-pilA*::Tn^37^ that lacks pili and thus twitching motility, was used as a negative control, and grown on TSA with tetracycline (60 µg/mL). We have previously verified this mutant^15^. Twitching motility assays involved growing bacteria on Twitching Motility Gellan Gum (TMGG) medium (0.8 g gellan gum, 0.4 g tryptone, 0.2 g yeast extract, 0.2 g NaCl, 0.1 g MgSO_4_·7H_2_O, in 100 mL H_2_O) at 37 °C in a humidified chamber for different times as specified, and as previously described^15^. For DMBT1 purification and agglutination assays (see below), we used *S. pyogenes* (ATCC19615) grown in Brain and Heart Infusion broth at 37 °C (overnight).

### Purification of DMBT1 from human saliva

Human saliva was obtained from healthy volunteers (4 subjects) using a protocol approved by the Committee for the Protection of Human Subjects, University of California, Berkeley. This research followed the tenets of the Declaration of Helsinki, and methods were performed in accordance with United States Federal guidelines and regulations. Informed consent was obtained from all subjects, and each was informed of the nature of the study and potential consequences. Collected saliva was first clarified by centrifugation (3,800 × g, 10 min). To purify DMBT1 we used methods described previously^7,38^. Firstly clarified saliva was diluted 50% in PBS. *S. pyogenes* grown as above, was also collected by centrifugation (3,800 × g, 5 min), then washed with PBS 3 times. After adjusting the bacterial concentration to ~5 × 10^9^ CFU/mL, equal volumes of bacterial suspension and diluted saliva were mixed and incubated (37 °C, 1 h). Bacteria were the collected by centrifugation (3,800 × g, 5 min), and washed with PBS 3 times. PBS (1.5 mL) with EDTA (5 mM) was used to release bound protein at room temperature (RT) (5 min). After centrifugation of the bacterial culture (15,000 × g, 5 min), the supernatant was passed through a 0.22 µm filter, and dialyzed (Slide-A-Lyzer dialysis cassettes, Thermo Fisher, NY) against PBS (4 °C, overnight). Dialyzed eluate was subjected to gel filtration chromatography on a Superose 6 10/300 GL column (GE Healthcare, CA) equilibrated in PBS (pH 7.4). Eluate at void volume was collected and used as purified DMBT1 (from saliva). DMBT1 concentration was measured with a micro BCA protein assay kit (Thermo Scientific, IL, USA).

### Twitching motility assays

Twitching motility was measured using a microscope slide assay^39^ with modifications previously described^15^. Bacteria were grown as described above, and TMGG medium dried for 20 min in a sterile airflow (BSL2 Biosafety Cabinet). Then, 5 µL of DMBT1, PBS or other solution was dropped onto the TMGG medium until absorbed. Bacteria were then inoculated onto the TMGG medium using a sterile toothpick. A glass coverslip was placed onto the medium to create an “interstitial” space. After incubating the slides for 4 h (37 °C), time-lapse videos (5 min) were captured at 10 s intervals *via* differential interference contrast (DIC) microscopy using a Nikon ECLIPS Ti microscope with a 60× oil-immersion objective at 37 °C.

### Quantification of twitching motility

To calculate twitching velocity, the twitching distance of a colony leading edge was divided by time as previously described^15^. Image J was used to measure the distance traveled by bacteria from frames 1–31 of the 5 min video. Ten bacteria were tracked in each video, and each group performed in triplicate.

### Dot-immunoblotting to test pili binding to DMBT1 or other solutions

Pili extracts were prepared as follows: *P. aeruginosa* PAO1 was suspended in PBS (OD_600_ of ~10), mixed by vortex (3 min), centrifuged (15,000 × g, 20 min) and the supernatant collected. After adding MgCl_2_ to the supernatant (to a final concentration of 100 mM), the supernatant was stored overnight at 4 °C. The next day, the superantant was centrifuged (15,000 × g, 20 min), and the pellet resuspended in PBS (500 μL) forming the pili-containing extract. PAO1-*pilA*::Tn was taken through the same process to make a negative control extract. Dot-immunoblot assays were performed as previously described^15^. Firstly, 2 μL of DMBT1 in PBS (250 ng/μL or 200 ng/μL, the latter concentration being equally effective) was spotted onto a nitrocellulose membrane (0.2 μm pore-size, BioRad). A PBS control was included along with pili extracts from PAO1 (positive control) or *pilA* mutant (negative control). The latter controls were prepared by diluting extracts in PBS (1 in 500). After drying, the membrane was blocked with BSA (5%) for 1 h at RT, washed for 5 min with PBS, then incubated with undiluted PAO1 pili extracts of PAO1 for 40 min at RT. After washing 5 times with PBS, membranes were probed with anti-PilA primary antibody (1:5000), then Goat anti-Rabbit HRP-conjugated secondary antibody (1:5000). Dot intensity was measured with AlphaView FluoChem HD2 software.

### Digestion of DMBT1 by Lys-C

Endoproteinase Lys-C (New England BioLabs) was used to digest the carboxyl side of lysine residues of DMBT1 under native conditions according to the manufacturer’s instructions. Then, 50 µL of purified DMBT1 (200 ng/µL) was incubated with 10 µL of the Lys-C digestion solution (100 ng/µL), 10 µL of PBS, and 2 µL of Lys-C solution (20 mM Tris-HCL buffer, pH 8.0) at 37 °C for 16 h. The sample was separated by size exclusion chromatography. An AKTAmicro system was used with a Superose 6 10/300 GL column (GE Healthcare) with elution in PBS (pH 7.4). Peak broadening was minimized by using short lengths of 0.15 mm i.d. tubing between the injection valve and fraction collector. Digested DMBT1 was injected onto the column, and fractions (250 μL) collected. Protein was detected by UV absorbance at 280 nm. Eluted fractions were pooled according to protein peaks and concentrated using a ~3 kDa cut-off filter (Millipore). Fraction effects on twitching motility were then assessed.

### SRCR peptides

Based on the amino acid sequence of DMBT1, a series of peptides spanning the consensus sequence of the DMBT1 SRCR domains (SRCRPs 1 to 7) were custom-synthesized as previously described^3^. Peptides were diluted to 200 ng/µL in Tris buffered Saline (TBS), pH = 7.5 supplemented with 10 mM calcium chloride and effects on bacterial twitching motility were tested as described above. For dot-immunoblots, 2 µL (400 ng) of SRCRPs 1–7 were spotted onto nitrocellulose membranes as described above.

### Agglutination assay

Agglutination assays were performed as previously described^3^. *S. pyogenes* and *P. aeruginosa* (PAO1) were suspended in TTC (Tris-Buffered Saline containing 0.1% Tween 20 and 1 mM Ca^2+^) to a concentration of ~5 × 10^8^ CFU/mL. Then, 150 µL of bacteria were mixed with 150 µL of SRCRP2 solution (200 ng/µL in Tris-Buffered Saline [TBS] with 10 mM Ca^2+^) in a 96-well plate then incubated for 2 h at 37 °C. After agglutination, 10 µL of the bacteria were transferred on a microscope slide. Bacteria were heat-fixed then stained with crystal violet solution and examined with light microscopy. Buffer alone (TBS with 10 mM Ca^2+^) and purified DMBT1 (200 ng/µL) were used as controls.

### Recombinant CD163

Human recombinant CellExp™ CD163 was obtained from BioVision, Inc. (Milpitas, CA, USA). The recombinant protein has a reported Mw of 135–140 kDa due to glycosylation. For use in experiments, CD163 was resuspended in PBS to a final concentration of 200 ng/µL. Effects on *P. aeruginosa* twitching motility were tested as above.

### Enzymatic deglycosylation

A deglycosylation enzyme mix (Biolabs) was used to digest DMBT1 under native conditions. For each enzyme (PNGase F, *O-*Glycosidase, Neuraminidase, β1-4 Galactosidase, β-N-acetylglycosaminidase), 5 µL was used to make the enzyme mix and incubated with 20 µL of purified DMBT1 (250 ng/µL) at 37 °C for 48 h. For complex de-O-glycosylation, 25 µL of purified DMBT1 (200 ng/µL) was incubated with 10X glycobuffer 2 (2 µL), enzyme mix (Neuraminidase, β1-4 Galactosidase, β-N-acetylglucosaminidase, and O-Glycosidase) (5 µL each) at 37 °C for 2 h. To remove N-linked glycans, 15 µL of purified DMBT1 (200 ng/µL) was incubated with 10X glycobuffer 2 (3 µL), PNGase F (5 µL) and H_2_O (7 µL) at 37 °C for 48 h.

### Statistical analysis

Data were expressed as a mean ± standard error of mean (SEM) unless otherwise stated. The significance of differences between multiple groups was assessed using a two-way ANOVA with Tukey’s multiple comparisons for post-hoc analysis, or using a one-way ANOVA with Tukey’s or Dunnett’s multiple comparisons. The Kruskal-Wallis test with Dunn’s multiple comparisons was used for nonparametric data. *P* values of less than 0.05 were considered significant.
